# Unilateral Pulmonary Agenesis and Gastric Duplication Cyst: A Rare Association

**DOI:** 10.1155/2013/608706

**Published:** 2013-06-17

**Authors:** Amir Halilbasic, Fahrija Skokic, Nesad Hotic, Edin Husaric, Gordana Radoja, Selma Muratovic, Nermina Dedic, Meliha Halilbasic

**Affiliations:** Department of Pediatric Surgery, Children's Hospital, University Clinical Center Tuzla, Trnovac bb, 75000 Tuzla, Bosnia and Herzegovina

## Abstract

Lung agenesis and gastric duplication cysts are both rare congenital anomalies. Gastric duplication cysts can present with nausea, vomiting, hematemesis, or vague abdominal pain. Unilateral pulmonary agenesis can present with respiratory distress which usually occurs due to retention of bronchial secretions and inflammations. We report the unique case of right pulmonary agenesis associated with gastric duplication cyst.

## 1. Introduction 

Lung agenesis is rare congenital anomaly, is often associated with acute respiratory distress, and has a high mortality rate [1]. Pulmonary agenesis implies the absence of a lung and its supporting vasculature, whereas the main bronchi may be either absent or hypoplastic [2]. Fifty percent born with pulmonary aplasia are stillborn or die within the first five years of  life. In unilateral lung agenesis, the trachea continues directly into the main bronchus of the normally developed lung, and respiratory distress usually occurs due to retention of bronchial secretions and inflammations [1]. Duplication of the alimentary tract is a relatively rare congenital anomaly. Duplication cysts of the stomach represent four per cent of all alimentary tract duplications [3]. When symptomatic, gastric duplication cysts can present with nausea, vomiting, hematemesis, or vague abdominal pain [4]. To our knowledge, we report the first case of a male newborn with right pulmonary agenesis associated with gastric duplication cyst.

## 2. Case Report

**Figure 1:**
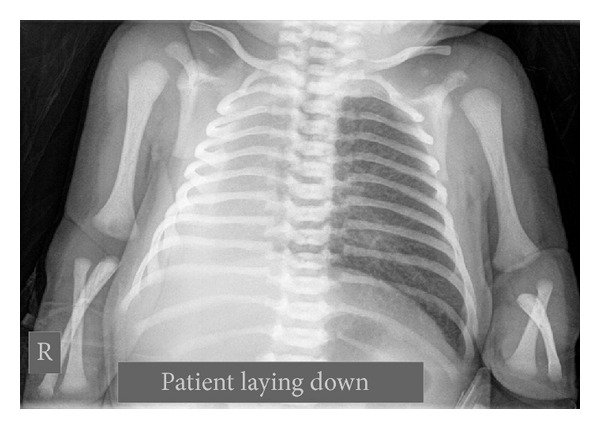
Opaque right hemithorax and left lung hyperinflation.

**Figure 2:**
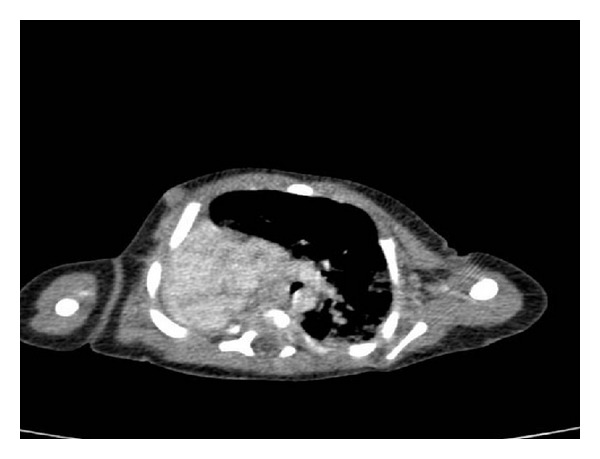
Absence of right main bronchus and right lung on CT scan.

Our patient was a male newborn, 4300 g, born at term by an uncomplicated vaginal delivery. The first- and five-minute Apgar score was 9/9. The mother had no significant past or obstetric history. On second day of life, the newborn became cyanotic with a respiratory rate up to 80/minute with chest retractions. There was absence of breath sounds in the right side of the chest. The heart sounds seemed loudest in the right chest. Her hematologic and other blood parameters were all normal. Posterior-anterior chest X-ray showed opaque right hemithorax with cardiac displacement and left lung hyperinflation (Figure 1). Contrast enhanced CT-scan thorax was done which showed absence of right bronchus and pulmonary parenchyma with normal hyperinflated left lung (Figure 2).

**Figure 3:**
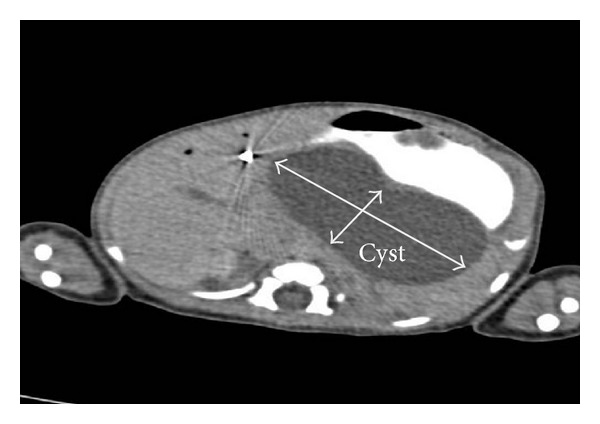
Cyst along posterior wall of stomach.

Echocardiogram showed ventricular septal defect and heart positioning in right hemithorax. Large cyst in upper abdomen measuring 6 × 2 centimeters was identified with abdominal ultrasound. Contrast enhanced abdominal CT scan verified bilobar cyst along posterior wall of stomach, 7.5 × 3.5 cm, with gastric compression (Figure 3). Cyst also compressed the pancreas and the spleen and it was in close contact with liver. 

During the first week of life, with supportive therapy, newborn was not oxygen dependent, tolerating feeding with normal bowel movement. On the 8th day of life the newborn rejected food and started with nonbilious vomiting. Because of gastric outlet obstruction, urgent surgical treatment was undertaken. Excision of large cyst formation was performed through the transversal supraumbilical laparotomy. Cyst shared common wall with stomach. Gastric wall defect was sutured with absorbable interrupted sutures. Histopathologically, the cyst had smooth muscles in the wall and inner lining of gastric mucosa. Patient's postoperative course was uneventful. The newborn was extubated 8 hours after surgery. No genetic tests were performed yet. Oral feeding resumed on the 4th postoperative day and it was discharged home with no respiratory symptoms. Follow-up period of four months after discharge was uneventful. 

## 3. Discussion

Congenital deficiency of the unilateral lung is believed to be caused by failure to maintain the developmental balance between the two lung buds [2]. Approximately, one in 15,000 children is born with a congenital absence of one lung. Associated malformations, mainly affecting the cardiovascular, gastrointestinal, and musculoskeletal systems, influence the prognosis of these patients, as well as the location of the missing lung [5]. There is a 1.3 : 1 female predominance with unilateral agenesis. Agenesis of the left lung appears to occur more frequently and the prognosis is generally regarded as worse for patients with agenesis on the right [2]. Our patient was male with right lung agenesis. Gastric duplication cyst is also rare congenital anomaly with incidence of roughly 17 on every 1,000,000 births. Approximately, 67 percent of gastric duplication cysts are identified within the first year of life [3]. The essential criteria for diagnosis of a gastric duplication cyst are (a) the wall of the cyst is contiguous with the stomach wall; (b) the cyst is surrounded by smooth muscle, which is continuous with the muscle of the stomach, and (c) the cyst wall is lined by epithelium of gastric or any other type of gut mucosa [6]. Many theories exist for the development of this lesion including a persistent embryological diverticulum, aberrant recanalization of the alimentary tract, partial twinning, and in utero ischemic events [4]. Anomalies that can be associated with unilateral pulmonary agenesis are esophageal atresia, tracheoesophageal fistula, tracheal stenosis, musculoskeletal anomalies, patent ductus arteriosus, and total anomalous pulmonary venous return [5]. Close to 50% of gastric cysts are associated with other abnormalities [7]. In our case, unilateral pulmonary agenesis was associated with gastric duplication cyst along with presence of ventricular septal defect. If symptomatic, gastric duplication is managed surgically by simple excision, by dissecting the common wall between the stomach and the duplication cyst, and usually it can be done easily without entering the stomach. But in cases where one does not get a plane of dissection in the common wall one should excise the common wall and suture the gastric defect, like we did in our case [8]. Extrapulmonary anomalies, as mentioned above, are frequently associated with pulmonary agenesis and worsen the prognosis, but even without other anomalies, children with isolated pulmonary agenesis have a shorter life expectancy than normal children, rarely surviving after their first decade [2]. In our case, rare association of rare anomalies did not have any impact on survival of newborn surgically treated for gastric outlet obstruction caused by large gastric duplication cyst. The finding of unilateral pulmonary agenesis associated with gastric duplication cyst and ventricular septal defect in our patient represents a unique case previously unreported.
